# Cytotoxicity of doxorubicin-conjugated poly[*N*-(2-hydroxypropyl)methacrylamide]-modified γ-Fe_2_O_3_ nanoparticles towards human tumor cells

**DOI:** 10.3762/bjnano.9.236

**Published:** 2018-09-25

**Authors:** Zdeněk Plichta, Yulia Kozak, Rostyslav Panchuk, Viktoria Sokolova, Matthias Epple, Lesya Kobylinska, Pavla Jendelová, Daniel Horák

**Affiliations:** 1 Institute of Macromolecular Chemistry CAS, Heyrovského nám. 2, 162 06 Prague 6, Czech Republic; 2 Department of Regulation of Cell Proliferation and Apoptosis, Institute of Cell Biology, NAS of Ukraine, Drahomanov Str. 14/16, Lviv 79005, Ukraine; 3 Inorganic Chemistry and Center for Nanointegration, University of Duisburg-Essen, Universitaetsstr. 5-7, D-45117 Essen, Germany; 4 Danylo Halytsky Lviv National Medical University, Pekarska Str. 69, Lviv 79000, Ukraine; 5 Institute of Experimental Medicine CAS, Vídeňská 1083, 142 20 Prague 4, Czech Republic

**Keywords:** cytotoxicity, doxorubicin, magnetic, nanoparticles, poly[*N*-(2-hydroxypropyl)methacrylamide]

## Abstract

Doxorubicin-conjugated magnetic nanoparticles containing hydrolyzable hydrazone bonds were developed using a non-toxic poly[*N*-(2-hydroxypropyl)methacrylamide] (PHPMA) coating, which ensured good colloidal stability in aqueous media and limited internalization by the cells, however, enabled adhesion to the cell surface. While the neat PHPMA-coated particles proved to be non-toxic, doxorubicin-conjugated particles exhibited enhanced cytotoxicity in both drug-sensitive and drug-resistant tumor cells compared to free doxorubicin. The newly developed doxorubicin-conjugated PHPMA-coated magnetic particles seem to be a promising magnetically targeted vehicle for anticancer drug delivery.

## Introduction

Severe side effects are considered to be the main drawback of conventional anticancer drugs. The drug dosage is significantly limited and thus complete elimination of tumor cells in cancer patients is not guaranteed. Among antitumor drugs, special attention has been paid to the anthracycline antibiotic doxorubicin (Dox), which is considered as one of the most potent FDA-approved antitumor drugs [1]. Dox realizes its therapeutic effect via the inhibition of DNA topoisomerase II and the generation of free radicals, leading to cell membrane damage, inhibition of macromolecule production, and ultimately induction of apoptosis [2–3]. However, its main shortcomings include dose-dependent cardio-, myelo-, and nephrotoxicity [4]. Moreover, Dox quickly disappears after intravenous administration from blood and concentrates in liver, kidneys, myocardium, spleen and lungs even if these organs are not the target of its actions. Novel approaches are therefore being developed to enhance the anticancer activity of Dox and decrease its side effects. Polymer-coated γ-Fe_2_O_3_ nanoparticles conjugate to Dox seem to be the most promising candidate for the role of such agents to achieve a high specificity and low side toxicity [5–6].

Superparamagnetic iron oxide nanoparticles with well-defined sizes, morphologies and surface are extremely useful in many different areas, in particular in biomedicine. As drug-delivery vehicles, such particles offer significant advantages compared to conventional drug formulations [7–9]. These include the presence of specific conjugated antibodies on the surface to bind selectively to related receptors and inhibit tumor growth and/or release loaded drugs for targeted therapy. This will result in increased drug concentration and accumulation at the pathological site, improved therapeutic performance of the anticancer agents and reduced non-specific toxicity to normal cells. Moreover, the strong magnetic susceptibility of the nanoparticles enables magnetic targeting, and the accumulation of these particles can be monitored by magnetic resonance imaging (MRI). Magnetic targeting is a minimally invasive method that increases the exposure of affected tissues to drug-loaded magnetic nanoparticles [5]. Colloidal stability, high drug-loading capacity, and relatively long circulation time are of primary importance for diverse biomedical applications of magnetic nanoparticles ranging from MRI contrast agents to drug-delivery systems, local heat sources in magnetic hyperthermia therapy of tumors, magnetically assisted transfection of cells, and magnetic field-assisted separation techniques. Let us to note that MRI is already widely used in human medicine and several iron-oxide-based contrast agents have been approved by the regulatory authorities.

It is well-known that the in vivo distribution of iron oxide nanoparticles is strongly influenced by their surface coatings. The coating ensures colloidal stability, prevents particles from aggregation, introduces functional groups for chemical attachment of target biomolecules, reduces cytotoxicity, and controls particle uptake by the cells [10]. Coatings can be either of low molecular weight, such are carbohydrates and organic acids (ethylenediaminetetraacetic acid, tartaric acid, citric acid, succinic acid, bisphosphonic acid), or of high molecular weight, such as silica, chitosan, poly(amino acids), poly(acrylic acid), hyaluronic acid, alginic acid, poly(vinyl alcohol), polyethylenimine, dextran, or poly(ethylene glycol) (PEG) [10–12]. The latter one is known to escape recognition by reticuloendothelial system prolonging thus blood circulation time of the particles; however, it is often immunogenic [13]. As a better alternative to PEG, poly[*N*-(2-hydroxypropyl)methacrylamide] (PHPMA) was suggested, which was also used as a substitute for blood plasma [14]. Nevertheless, it has to be noted that nanotechnology in drug development is at its early stage, transfer to clinical environments is problematic, and improvement of the efficacy–toxicity balance is required [15].

In this report, we took advantage of the versatility of PHPMA as a base for iron-oxide coating. The objective is to investigate the in vitro cytotoxic activity of Dox-conjugated poly[*N*-(2-hydroxypropyl)methacrylamide-*co*-2-(*N*-methylmethacrylamido)acetate] [P(HPMA-MMAA)]-coated magnetic γ-Fe_2_O_3_ particles [γ-Fe_2_O_3_@P(HPMA-MMAA)-Dox] as a prospective vehicle for the transport of anticancer drug into cells. To the best of our knowledge, polymers based on reactive methyl ester sarcosine derivatives were not yet described as a promising coating of magnetic nanoparticles. The cytotoxic behavior of the nanoparticles with immobilized Dox was investigated in different models of murine and human tumor cell lines and compared with Dox itself and PHPMA-coated nanoparticles (without Dox). We have chosen the HeLa immortal cell line derived from cervical cancer, human T-leukemia cells Jurkat, K562, HL-60/wt and its drug-resistant HL-60/vinc subline, the highly metastatic murine B16F10/wt melanoma cell line, and the human osteosarcoma MG63 cell line. As a model of human undifferentiated cells, mesenchymal stem cells were utilized. Cellular uptake of agents was studied by fluorescence microscopy and induction of cell death was visualized by live/dead assay. Dox-conjugated γ-Fe_2_O_3_@P(HPMA-MMAA) particles showed enhanced cytotoxicity in drug-sensitive and drug-resistant tumor cells.

## Experimental

### Materials

Sarcosine methyl ester hydrochloride, 4-cyano-4-(phenylcarbonothioylthio)pentanoic acid (CTPA), 4′,6-diamidino-2-phenylindole (DAPI), dimethyl sulfoxide (DMSO), DMEM and RPMI medium supplemented with 10% fetal calf serum streptomycin and penicillin were from Sigma-Aldrich (St. Luis, MO, USA). Methacryloyl chloride (Sigma-Aldrich) was distilled and 2,2′-azobis(2-methylpropionitrile) (AIBN; Fluka; Buchs, Switzerland) was recrystallized from ethanol. Doxorubicin hydrochloride (Dox) was from TCI Chemicals (Tokyo, Japan). Phosphate buffered saline (PBS) was prepared on site. 1,2-Dichloroethane (DCE), hydrazine hydrate, ethanol (99.8%), and other chemicals were purchased from Sigma-Aldrich or Lach-Ner (Neratovice, Czech Republic) and used as received. *N*-(2-hydroxypropyl)methacrylamide (HPMA) and citrate-stabilized maghemite (γ-Fe_2_O_3_) were synthetized according to earlier procedures [16–17].

#### Synthesis of methyl 2-(*N*-methylmethacrylamido)acetate (MMAA)

**Figure 1:**
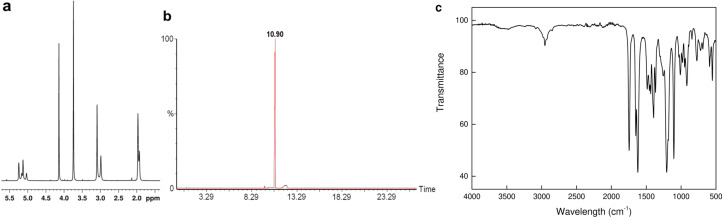
(a) ^1^H NMR, (b) gas chromatogram, and (c) FTIR spectrum of 2-(*N*-methylmethacrylamido)acetate (MMAA).

In a 250 mL reaction flask, sarcosine methyl ester hydrochloride (14 g; 0.1 mol), triethylamine (20.2 g; 0.2 mol), and methacryloyl chloride (10.6 g; 0.1 mol) were dissolved in DCE (100 mL) under stirring (800 rpm) at 50 °C for 20 min and under Ar atmosphere. Methanol (3 mL) was added to transform unreacted methacryloyl chloride in methyl methacrylate and the mixture was stirred until reaching room temperature (RT). Precipitated triethylamine·HCl was filtered-off, DCE removed on a rotary evaporator, and the resulting MMAA was diluted in ethyl acetate (50 mL). Residual compounds and ethyl acetate were removed by filtration and vacuum-evaporation, respectively, and MMAA was distilled (100 °C/13 Pa); yield 55%. ^1^H NMR (CDCl_3_) δ 1.96 (s, 3H), 3.09 (s, 3H), 3.77 (s, 3H), 4.14 (s, 2H), 5.03 (dd, H), 5.24 (dd, H) (Figure 1a).

#### Reversible addition–fragmentation chain transfer (RAFT) copolymerization of HPMA with MMAA and preparation of hydrazide derivative of P(HPMA-MMAA)

In a 50 mL Erlenmeyer flask, CTPA (4 mg), AIBN (5 mg), MMAA (0.1 mL), and HPMA (0.9 g) were dissolved in ethanol (9 mL) under Ar atmosphere, 5 M HCl (50 μL) was added and the polymerization was started by heating at 60 °C for 16 h. Ethanol (5 mL) was removed on a rotary evaporator, the resulting poly[*N*-(2-hydroxypropyl)methacrylamide-*co*-2-(*N*-methylmethacrylamido)acetate] [P(HPMA-MMAA)] was precipitated in diethyl ether, separated by centrifugation, repeatedly washed with diethyl ether, and dried; yield: 0.6 g.

To transform the polymer to its hydrazide derivative, P(HPMA-MMAA) (0.6 g) was dissolved in ethanol (2 mL) in a 25 mL beaker, hydrazine hydrate (50 μL) was added, and the reaction continued at RT for 16 h with stirring (6 rpm). The viscous P(HPMA-MMAA)-NH-NH_2_ was diluted with ethanol (2 mL)/tetrahydrofuran (4 mL) mixture and precipitated in diethyl ether (10 mL) and dried. Finally, to attach Dox, P(HPMA-MMAA)-NH-NH_2_ (40 mg) was dissolved in methanol (0.3 mL), a solution of Dox (3.9 mg) in methanol (1 mL) and 0.5 M methanolic acetic acid (25 µL) was added, and the reaction continued at RT for 16 h. Ethanol (1 mL) was added and the resulting P(HPMA-MMAA)-Dox was precipitated twice in ethyl acetate (5 mL), separated by centrifugation (5000 rpm), and dried in air. The amount of DOX in P(HPMA-MMAA)-Dox was determined spectrophotometrically.

#### Coating of γ-Fe_2_O_3_ with PHPMA and P(HPMA-MMAA)-Dox

First, PHPMA was prepared by precipitation polymerization and used as a coating of γ-Fe_2_O_3_ nanoparticles. Briefly, in an 100 mL Erlenmeyer flask, HPMA (2 g) freshly crystalized from ethyl acetate and AIBN (10 mg) were dissolved in ethyl acetate (18 mL) under Ar atmosphere and the polymerization proceeded at 60 °C for 16 h. The resulting PHPMA was washed with ethyl acetate and separated by filtration yielding 1.92 g of the polymer (*M*_n_ = 177 kDa). Aqueous solution of PHPMA (2 mL; 0.1 g PHPMA/mL) was then added to the γ-Fe_2_O_3_ colloid (2 mL; 0.1 g of γ-Fe_2_O_3_/mL) to obtain γ-Fe_2_O_3_@PHPMA. Second, P(HPMA-MMAA)-Dox (0.1–13.0 µg) was dissolved in aqueous γ-Fe_2_O_3_@PHPMA colloid (2–260 µg; 50 mg γ-Fe_2_O_3_/mL) to get γ-Fe_2_O_3_@P(HPMA-MMAA)-Dox before use in the cell experiments.

### Characterization methods

^1^H NMR (in CDCl_3_) and FTIR spectra of MMAA were recorded on a Bruker DPX 300 spectrometer (Billerica; MA, USA) and a Perkin Elmer PARAGON 1000 PC spectrometer (Bucks, UK), respectively. Gas and HPLC SEC chromatography was performed on a Clarus 500 Perkin Elmer gas chromatograph (Shelton, CT, USA) and a Shimadzu HPLC chromatograph (Kyoto, Japan) equipped with a mass spectrometry and UV–vis, RID, or DAWN 8 MALS detectors (Wyatt Technology; Santa Barbara, CA, USA), respectively. Agilent DB-35MS GC column (30 m × 0.25 mm id, 0.25 µm film; helium carrier gas) and Chromolith RP-18 e HPLC column (100 mm × 4.6 mm id) with a flow rate of 5 mL acetonitrile/water mixture per min were used. Number- (*D*_n_) and weight-average particle diameter (*D*_w_) and polydispersity index (PDI = *D*_w_/*D*_n_) characterizing the particle size distribution were determined from analysis of at least 500 particles on micrographs from a FEI Tecnai G^2^ Spirit transmission electron microscope (TEM; Brno, Czech Republic). Dynamic light scattering (DLS) was measured on a Zetasizer Nano-ZS Model ZEN3600 (Malvern Instruments; Malvern, UK) providing hydrodynamic diameter *D*_h_ and polydispersity PI. UV–vis spectra were recorded on a Specord 250 Plus spectrophotometer (Analytic Jena; Jena, Germany).

### Cytotoxicity studies

Mouse melanoma cells of B16F10/wt line, human T-leukemia cells of Jurkat, K562, HL-60 lines and its drug-resistant HL-60/vinc sub-line (overexpression of P-glycoprotein) were a kind gift of Prof. Walter Berger, Institute of Cancer Research, Vienna Medical University (Austria). Human mesenchymal stem cells (hMSCs), human cervix carcinoma cells of HeLa line and human osteosarcoma cells (MG-63) were obtained from cell culture collection of the University of Duisburg-Essen. MG-63 cells were cultured in DMEM medium, supplemented with 10% fetal calf serum (FCS), 100 U/mL penicillin, and 100 mg/mL streptomycin at 37 °C in humidified atmosphere containing 5% CO_2_, while hMSCs were cultured in mesenchymal stem cell growth medium (MSCGM BulletKit^TM^; Lonza, Italy). All other cells were cultured in RPMI medium supplemented with 10% fetal calf serum, streptomycin (50 µg/mL), and penicillin (50 units/mL) at 37 °C in humidified atmosphere containing 5% CO_2_.

In cell experiments, 10^6^ of Jurkat, K562, HL-60/wt, and HL-60/vinc cells, or 10^5^ of B16F10/wt cells were seeded in 24-well tissue culture plates (Greiner Bio-One; Kremsmünster, Austria). Short-term (24 h) cytotoxic effect of the particles was studied under Evolution 300 Trino microscope (Delta Optical; Mińsk Mazowiecki, Poland) after cell staining with 0.1% trypan blue. For long-term (72 h) cytotoxicity experiments, MTT assay was used. hMSC, HeLa, and MG-63 cells were plated (5 × 10^3^) in 100 µL of medium per well in 96-well plates and allowed to grow for 24 h. The particles dispersed in another 100 µL of culture medium were added (Table 1) and the incubation continued for 24, 48, or 72 h. MTT (3-(4,5-dimethylthiazol-2-yl)-2,5-diphenyltetrazolium bromide; Sigma-Aldrich) was dissolved in PBS (5 mg/mL) and then diluted to 1 mg/mL in cell culture medium.

**Table 1:** Concentration of Dox and polymer-modified iron oxide particles used in the MTT test.

sample	concentration (µg/200 µL)			

Dox	0.028	0.056	0.139	0.277
γ-Fe_2_O_3_@PHPMA	0.056	1.12	2.78	5.54
γ-Fe_2_O_3_@P(HPMA-MMAA)-Dox	0.056	1.12	2.78	5.54

The culture medium above the incubated cells was replaced by the MTT solution (300 µL) and the incubation continued for 1 h at 37 °C under 5% CO_2_ in humidified atmosphere. DMSO (300 µL) was added to the cells and after 30 min, a 100 µL aliquot was taken for spectrophotometric analysis with a Multiscan FC instrument (Thermo Fisher Scientific; Vantaa, Finland) at 570 nm. The absorption of solution above the incubated cells was normalized to that of control (untreated) cells, indicating the relative level of cell viability. Each concentration of the studied compounds was run in triplicate and normalized to blank controls, containing the equivalent volume of culture medium. Cytotoxicity was expressed as IC_50_ value calculated from full dose-response curves as drug concentration inducing 50% reduction in cell survival compared to the control cultured in parallel without the particles.

The uptake studies of Dox-conjugated polymer-coated γ-Fe_2_O_3_ nanoparticles by primary cells (hMSCs) and human tumor cells (MG-63 and HeLa) were carried out as follows. The cells were incubated with appropriate amounts of the particle colloids for 48 h and washed with PBS three times to remove dissolved compounds not attached to the cells. The cellular uptake was measured by fluorescence microscopy with a Keyence Biorevo BZ-9000 instrument (Osaka, Japan) equipped with filters for Texas Red (EX 560/40, DM 585, BA 630/75) at 20× magnification. Dox and its complexes were visible as red fluorescing dots.

Live/dead assay was carried out according to the following protocol. 72 h after the incubation of MG-63 and HeLa cells with γ-Fe_2_O_3_@PHPMA nanoparticles (2.78 µg), Dox (0.139 µg), and γ-Fe_2_O_3_@P(HPMA-MMAA)-Dox (2.78 µg γ-Fe_2_O_3_@PHPMA + 0.139 µg P(HPMA-MMAA)-Dox), the cells were washed with PBS and stained with a live/dead viability/cytotoxicity assay for mammalian cells (Invitrogen; Carlsbad, CA, USA) to evaluate the cell viability. Calcein AM and ethidium homodimer-1 (EthD-1) working solution (150 μL) was added to the cells, which were subsequently incubated at 37 °C for 30 min and imaged by a Keyence Biorevo BZ-9000 fluorescence microscope. The live/dead kit determined the cell viability based on the cell membrane integrity. Living cells were stained by calcein AM, which emits green fluorescence (517 nm) after excitation by blue light (494 nm), whereas dead cells were stained by EthD-1, which emits red fluorescence (617 nm) after excitation by green light (528 nm). All experiments were carried out in triplicate.

Finally, DAPI staining was used for determination of chromatin hypercondensation in the B16 melanoma cells treated with the Dox and its complexes for 24 h. After the treatment with γ-Fe_2_O_3_@PHPMA nanoparticles (2.78 µg), Dox (0.139 µg), and γ-Fe_2_O_3_@P(HPMA-MMAA)-Dox (2.78 µg γ-Fe_2_O_3_@PHPMA + 0.139 µg P(HPMA-MMAA)-Dox), the cells were washed with 1× PBS twice, fixed in 4% paraformaldehyde at RT for 15 min and permeabilized with 0.1% Triton X-100 in PBS for 3 min. Then, the cells were incubated with DAPI (1 µg/mL) for 5 min, washed with PBS twice, and fixed on a cover glass. Nuclear morphology was observed on a Carl Zeiss AxioImager A1 fluorescent microscope (Göttingen, Germany). Cells without the particles and/or Dox served as a negative control.

## Results and Discussion

### Poly[*N*-(2-hydroxypropyl)methacrylamide-*co*-2-(*N*-methylmethacrylamido)acetate] [P(HPMA-MMAA)] and doxorubicin (Dox) conjugation

**Figure 2:**
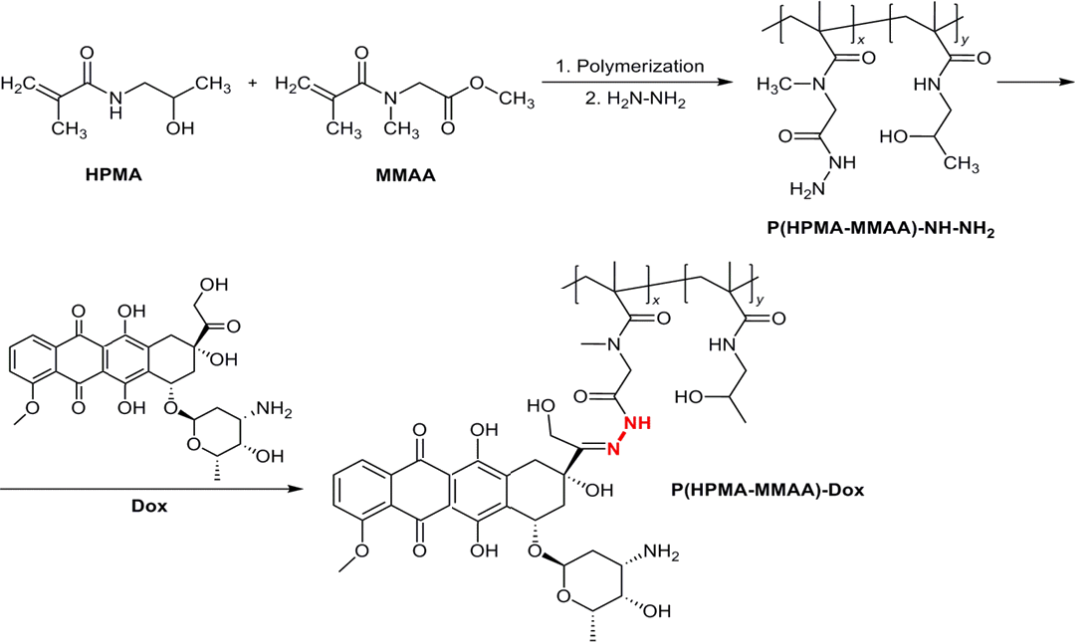
Scheme of RAFT copolymerization of *N*-(2-hydroxypropyl)methacrylamide (HPMA) with methyl 2-(*N*-methylmethacrylamido)acetate (MMAA), preparation of its hydrazide derivative, and reaction of P(HPMA-MMAA)-NH-NH_2_ with Dox.

In this report, HPMA and MMAA monomers were used for preparation of polymer coating for the γ-Fe_2_O_3_ nanoparticles (Figure 2).

The PHPMA backbone brings the benefits of hydrophilicity, hydrolytic stability, biocompatibility, absence of toxicity, minimal immunogenicity, and enhanced permeability and retention to cancer cells [18]. Moreover, PHPMA, which has a long history of biomedical applications as a drug-delivery vehicle, enables the control of biodistribution and accumulation via molecular weight limitations [19–20]. In contrast, MMAA introduces to the copolymer the methoxy functional group, which can be subsequently easily transformed to the hydrazide required for conjugation with compounds containing reactive carbonyl groups (aldehyde or ketone), such as Dox. To prepare the MMAA monomer, the reactive methyl ester of sarcosine was methacryloylated. Structure and purity of MMAA was confirmed by ^1^H NMR (Figure 1a), gas chromatography (Figure 1b), and FTIR spectroscopy (Figure 1c). The FTIR spectrum exhibited characteristic peaks at 1745 and 1620 cm^−1^ ascribed to strong C=O stretching vibrations of ester and amide groups of MMAA, respectively. Peaks at 3083, 1649, and 889 cm^−1^ belonged to the CH_2_=, C=C stretching, and CH_2_= wagging vibrations of methacrylate, respectively. A strong asymmetric stretching vibration of C–O–C appeared at 1206 cm^−1^, while bending vibrations at 1454 and 1367 cm^−1^ were attributed to CH_3_ and CH_2_ groups of MMAA. Bands at ca. 2900 and at 1500–1350 cm^−1^ corresponded to stretching and bending vibrations of CH_2_ and CH_3_ groups, respectively.

**Figure 3:**
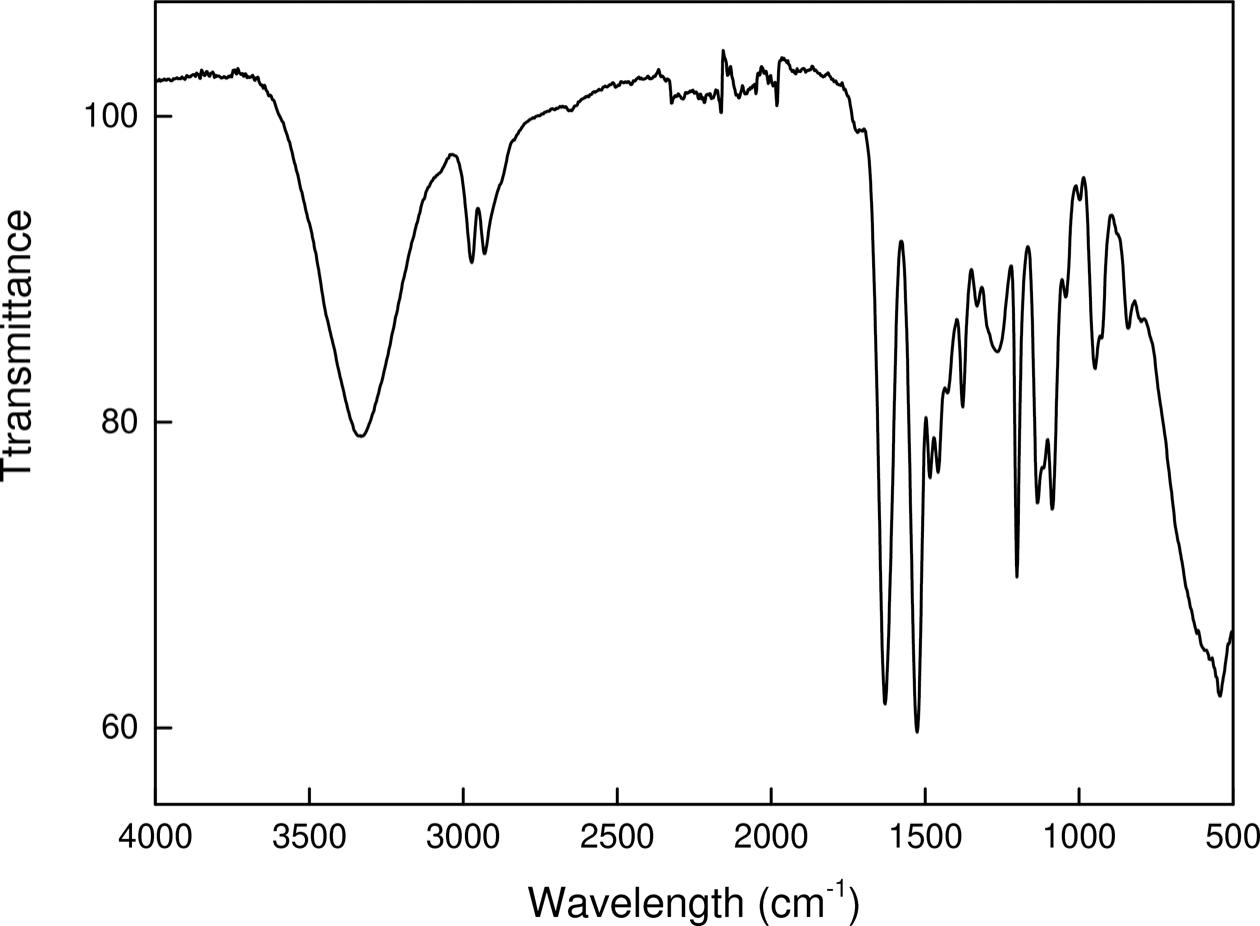
FTIR spectrum of poly[*N*-(2-hydroxypropyl)methacrylamide-*co*-2-(*N*-methylmethacrylamido)acetate] [P(HPMA-MMAA)].

The first polymerization experiments involved the precipitation copolymerization of HPMA and MMAA. High molecular weight products (ca. 170 kDa according to HPLC) were obtained, which was unacceptable from the point of in vivo biomedical applications. Therefore, RAFT copolymerization was selected, which enables the preparation of well-defined telechelic P(HPMA-MMAA) molecules of medium or low molecular weight [21]. With this technique the molecular weight of PHPMA can be kept below the renal threshold (*M*_w_ < 50 kDa), which allows for rapid renal clearance and avoids accumulation in the body. Indeed, the molecular weight of P(HPMA-MMAA) according to HPLC SEC chromatography was moderate (*M*_n_ = 33 kDa) with polydispersity index = 1.36. Formation of the copolymer was confirmed by the FTIR spectrum exhibiting characteristic peaks at 2973 and 2933 cm^−1^ ascribed to CH stretching vibrations. The peak at 1729 cm^−1^ belongs to C=O ester stretching vibrations and those at 1631 and 1528 cm^−1^ were attributed to C=O and NH amide stretching and bending vibrations, respectively (Figure 3).

The methoxy groups of the P(HPMA-MMAA) chains were reacted with hydrazine hydrate (Figure 2) under a rather low molar excess of hydrazine relative to MMAA in order to facilitate purification of the product. Finally, P(HPMA-MMAA)-NH-NH_2_ was conjugated with Dox in methanol and precipitated. Doxorubicin was attached to the polymer via hydrolyzable hydrazone bonds. According to UV–vis spectrometry, 30 mg of Dox was bound per gram of P(HPMA-MMAA).

### γ-Fe_2_O_3_ nanoparticles

**Figure 4:**
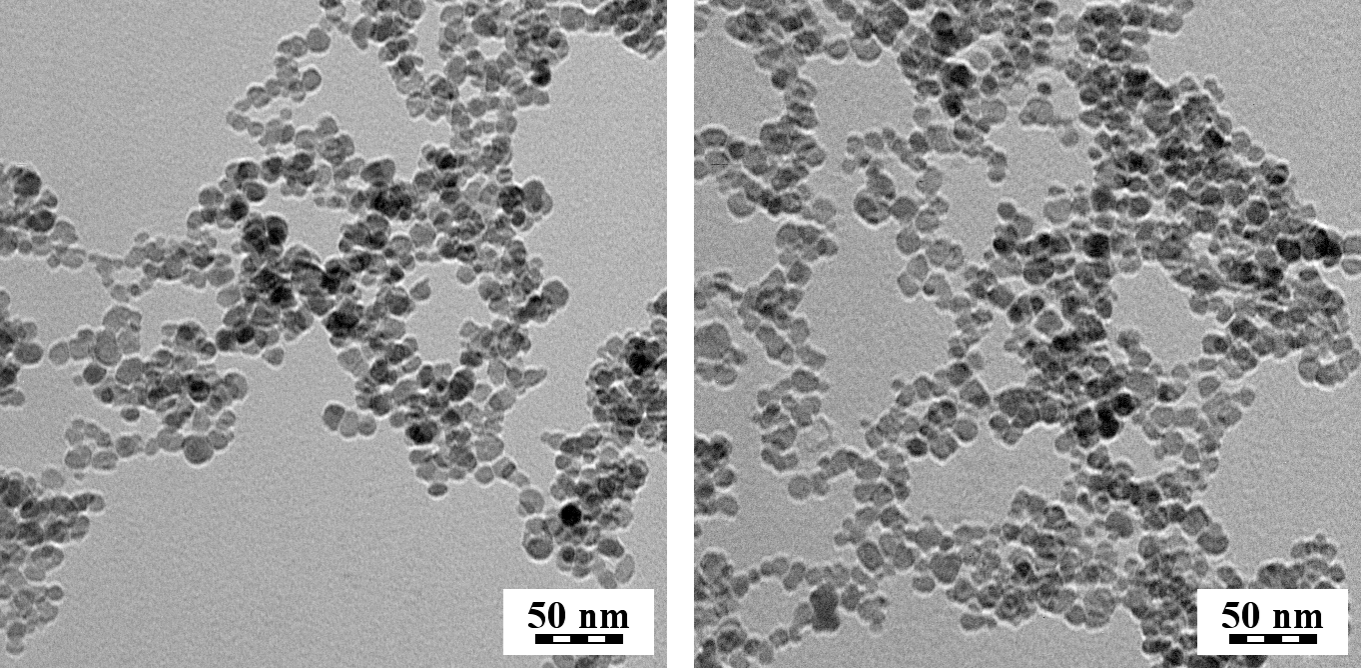
TEM micrographs of (a) γ-Fe_2_O_3_ and (b) γ-Fe_2_O_3_@PHPMA nanoparticles.

Coprecipitation and oxidation were selected for the synthesis of the magnetic γ-Fe_2_O_3_ nanoparticles, since they yield hydrophilic particles that are mildly polydisperse and do not need any transfer in water. The advantage of maghemite over magnetite consists in its chemical stability [22]. Moreover, the particles exhibited superparamagnetic behavior as documented in our previous paper [23]. This ensures their easy magnetic separation and at the same time redispersibility in the absence of magnetic fields, which is important for biomedical applications. According to the TEM micrograph of the dried γ-Fe_2_O_3_ particles, their shape was spheroid, with a diameter of *D*_n_ ≈ 10 nm and polydispersity index PDI = 1.23 documenting a moderately broad particle size distribution (Figure 4a).

The hydrodynamic diameter *D*_h_ of γ-Fe_2_O_3_ particles in water according to DLS was 90 nm with polydispersity PI = 0.15, which was in agreement with PDI. It is a general observation that *D*_h_ > *D*_n_, as DLS observes also solvent molecules associated with the particles. DLS is an intensity-based technique putting emphasis on the larger particles due the fact that the scattering intensity is proportional to the 6th power of the size, while TEM is a number-based observation. Moreover, small aggregates are also responsible for relatively high values of *D*_h_. The ζ-potential of the particles amounted to −35 mV (pH 7.3), which is sufficient for short-term colloidal stability of the particles.

### Coating of γ-Fe_2_O_3_ nanoparticles with poly[*N*-(2-hydroxypropyl)methacrylamide] (PHPMA)

**Figure 5:**
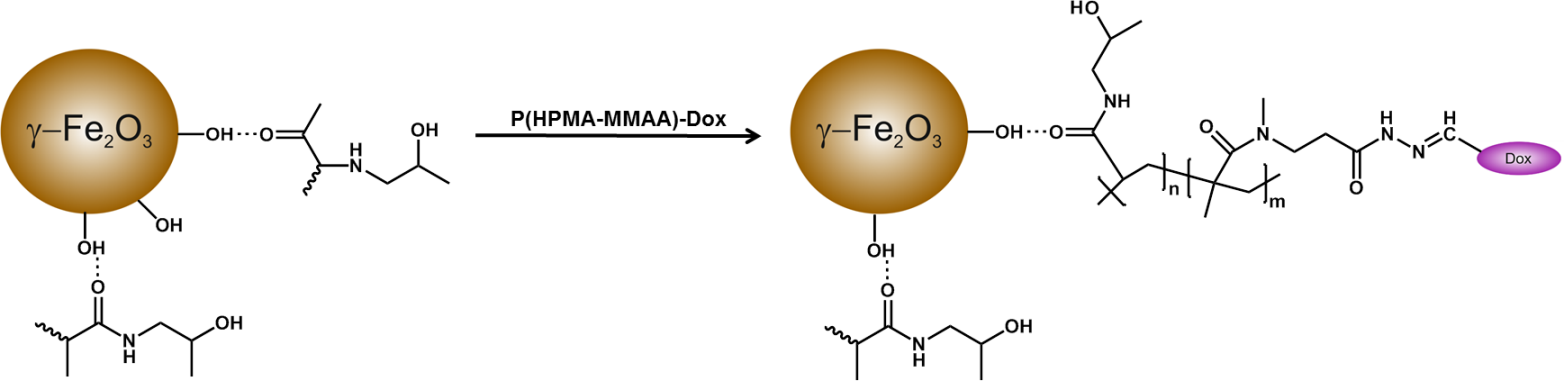
Schematic illustration of the interaction between PHMPA and a maghemite nanoparticle followed by the addition of P(HPMA-MMAA)-Dox.

Surface modification of nanoparticles is a general strategy for enhancing the long-term colloidal stability and the permeability of cells to nanoparticles. In this report, the surface of the γ-Fe_2_O_3_ nanoparticles was modified with PHPMA (*M*_n_ = 177 kDa) by a post-synthesis method. It is supposed that carbonyl groups of PHPMA bind to γ-Fe_2_O_3_ and interact with hydroxy groups on the iron oxide surface (Figure 5).

According to TEM, the PHPMA coating changed neither the morphology nor the size and polydispersity of the particles (Figure 4b). PHPMA itself was not visible on the TEM micrographs due to its low electron density. The hydrodynamic diameter of γ-Fe_2_O_3_@PHPMA (*D*_h_ = 165 nm; PI = 0.22) was substantially larger than that of γ-Fe_2_O_3_ (90 nm). This is due to the fact that the hydrodynamic diameter gives information of the γ-Fe_2_O_3_ core along with coating material and the solvent layer attached to the particles, which undergo Brownian motion. Moreover, clustering of the γ-Fe_2_O_3_@PHPMA particles also contributes to increased values of *D*_h_. The ζ-potential of γ-Fe_2_O_3_@PHPMA substantially differed from that of γ-Fe_2_O_3_, reaching +38 mV and indicating the presence of coating. After mixing γ-Fe_2_O_3_@PHPMA nanoparticle colloid with P(HPMA-MMAA)-Dox, its chains readily intertwine with the PHMPA corona chains and adsorb to the γ-Fe_2_O_3_ particle surface (Figure 5). The rather large hydrodynamic size enables easy magnetic separation from the medium.

### Cytotoxicity of γ-Fe_2_O_3_@P(HPMA-MMAA)-Dox nanoparticles

**Figure 6:**
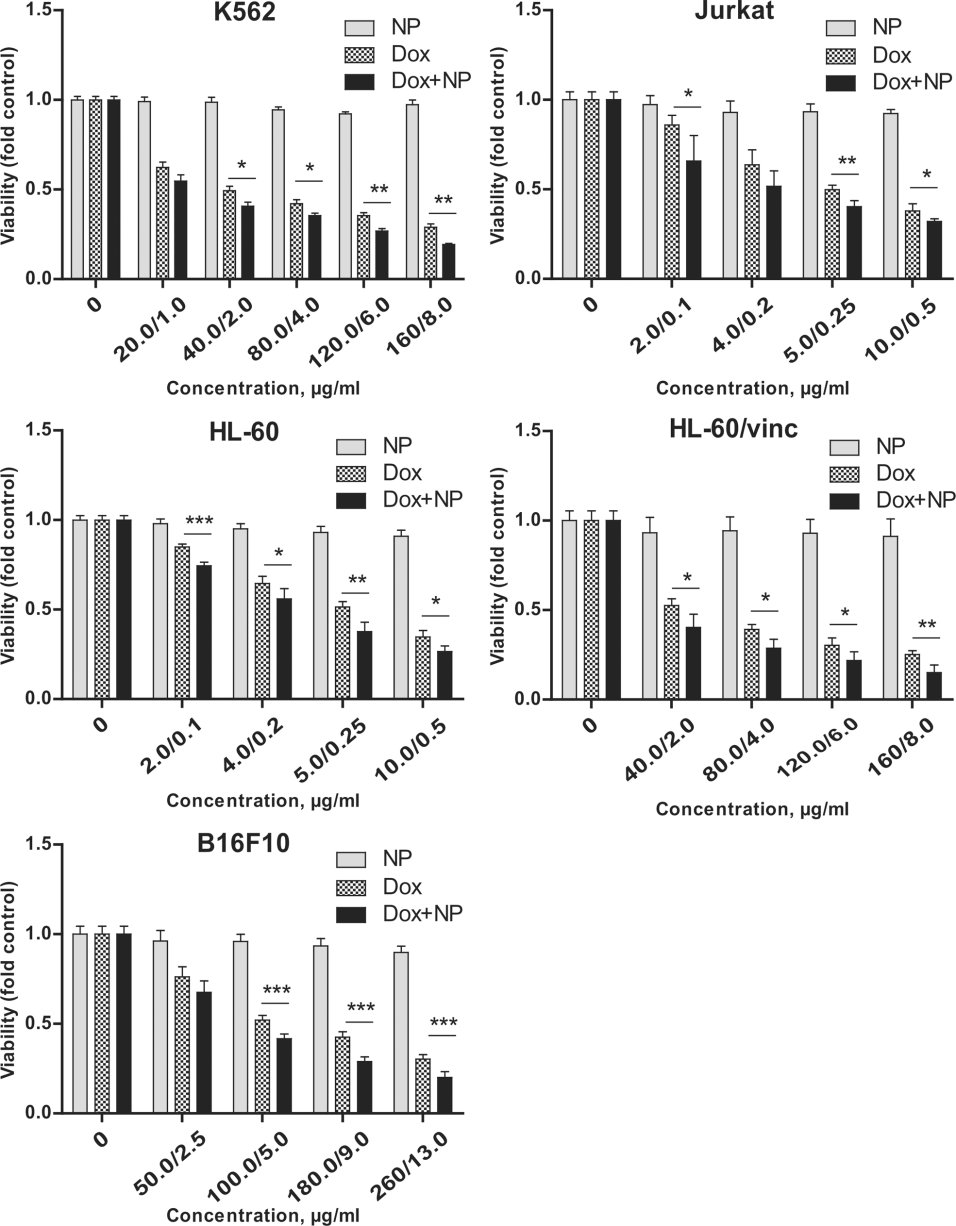
Comparison of short-term cytotoxicity (24 h of incubation) of γ-Fe_2_O_3_@PHPMA (NP), Dox, and γ-Fe_2_O_3_@P(HPMA-MMAA)-Dox nanoparticles (Dox+NP) towards human leukemia cells of K562, Jurkat, HL-60/wt and HL-60/vinc lines (seeded 10^6^ per mL) and murine melanoma cells of B16F10/wt line (seeded 10^5^ per mL); trypan blue staining. Data are relative to the untreated controls and represent the mean +/− SD of three independent experiments. * *p* < 0.05 relative to Dox, ** *p* < 0.01 relative to Dox, *** *p* < 0.0001 relative to Dox, unpaired t-test. Significance levels indicated above bars refer to the comparison with the respective Dox-treated controls.

To study antitumor activity of the γ-Fe_2_O_3_@P(HPMA-MMAA)-Dox nanoparticles, various cancer cell lines (Jurkat, K562, HL-60/wt, HL-60/vinc, and B16F10/wt) were treated with the nanoparticles and their cytotoxic activity was compared to that of free Dox (Figure 6).

**Figure 7:**
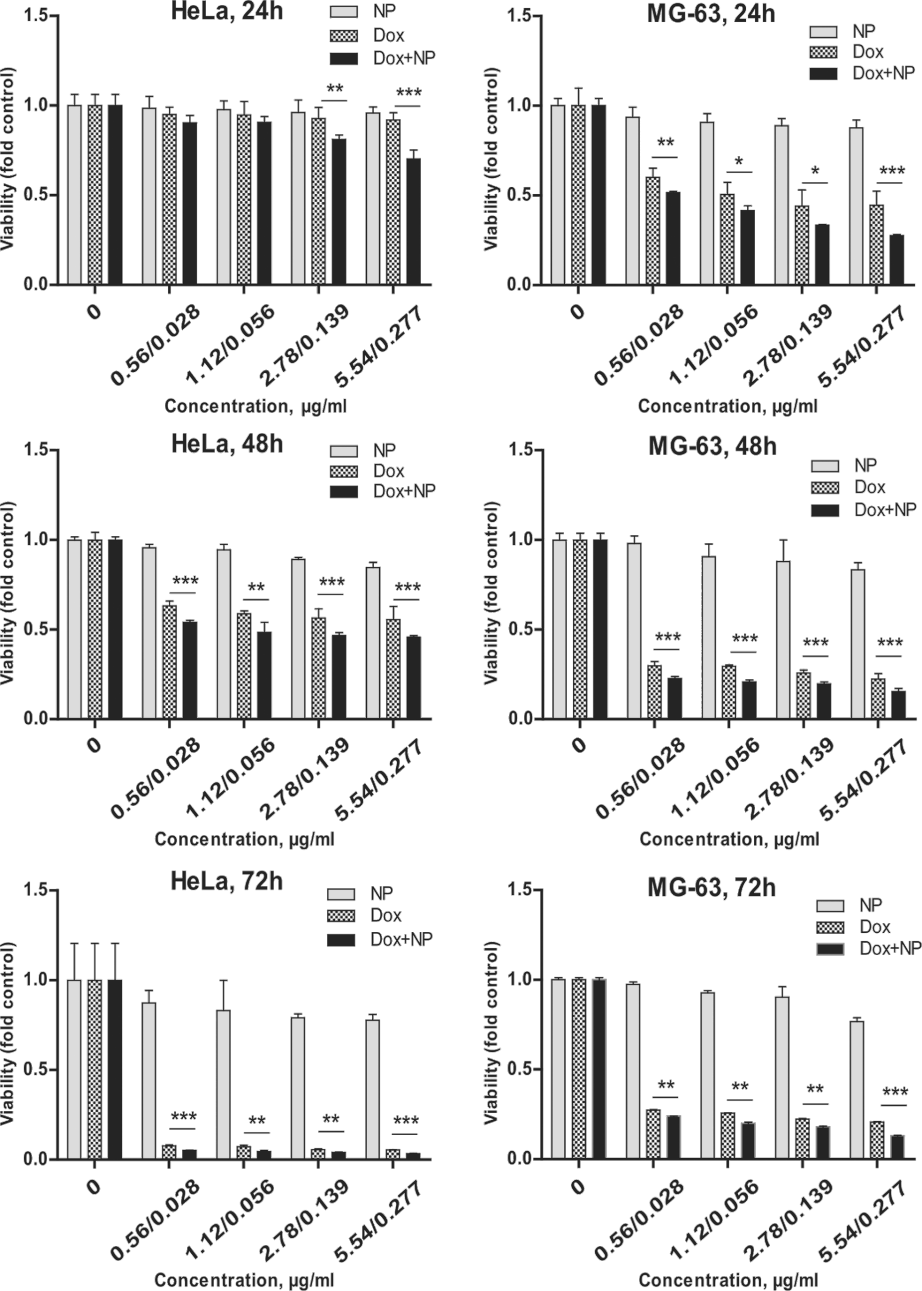
Comparison of short- and long-term cytotoxicity of γ-Fe_2_O_3_@PHPMA (NP), Dox, and γ-Fe_2_O_3_@P(HPMA-MMAA)-Dox nanoparticles (Dox+NP) towards human cervix carcinoma cells of HeLa line and human osteosarcoma cells of MG-63 line (seeded 5∙10^3^ per 100 µL), MTT assay. Agents were added in 200 µL of medium. Data are relative to the untreated controls and represent the mean +/− SD of three independent experiments. * *p* < 0.05 relative to Dox, ** *p* < 0.01 relative to Dox, *** *p* < 0.0001 relative to Dox, unpaired t-test. Significance levels indicated above bars refer to the comparison with the respective Dox-treated controls.

**Figure 8:**
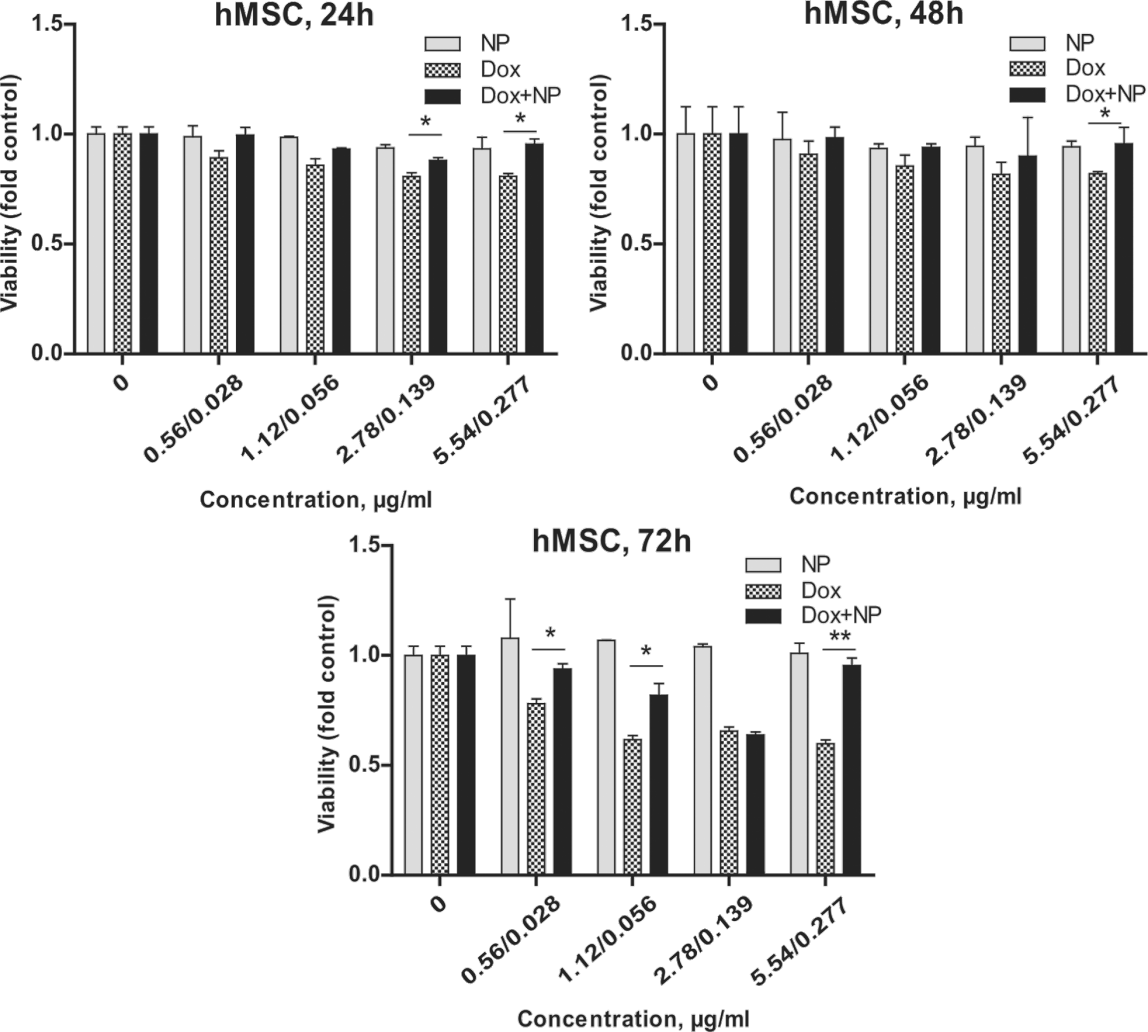
Comparison of short- and long-term cytotoxicity of γ-Fe_2_O_3_@PHPMA (NP), Dox, and γ-Fe_2_O_3_@P(HPMA-MMAA)-Dox nanoparticles (Dox+NP) towards human mesenchymal stem cells (hMSC, seeded 5∙10^4^ per mL), MTT assay. Data are relative to the untreated controls and represent the mean +/− SD of three independent experiments. *p < 0.05 relative to Dox, ** *p* < 0.01 relative to Dox, unpaired t-test. Significance levels indicated above bars refer to the comparison with the respective Dox-treated controls.

γ-Fe_2_O_3_@P(HPMA-MMAA) nanoparticles used in different amounts (2–260 μg depending on cell line) were non-toxic for all these cell lines. γ-Fe_2_O_3_@P(HPMA-MMAA)-Dox nanoparticles demonstrated slightly higher toxicity (by 5–10%) towards human leukemia cells of Jurkat and HL-60/wt lines compared to free Dox, while drug-resistant HL-60/vinc cells, overexpressing P-glycoprotein, demonstrated a significantly higher sensitivity to the action of these nanoparticles, inducing 20% cell death. Such specific action of Dox-conjugated nanoparticles may be explained by targeted delivery of Dox to tumor cells. It can be supposed that the slow release of Dox from the nanoparticle surface due to the hydrolysis of hydrazone bonds allows for a stable Dox concentration inside the cells, thus partially decreasing the effectiveness of ABC transporter proteins, which are responsible for drug efflux from the cytosol to the extracellular medium [24]. Other cell lines that are sensitive to chemotherapy (e.g., murine B16F10 melanoma, human K562 leukemia) demonstrated a similar sensitivity to Dox-conjugated nanoparticles; cell number decreased by 3–10% (Figure 6). Additionally, the long-term effect (72 h) of these nanoparticles was studied towards hMSCs, human MG-63, and HeLa tumor cells by MTT assay and compared to that of free Dox (Figure 7 and Figure 8).

**Figure 9:**
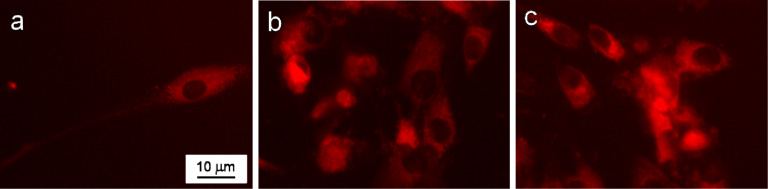
Fluorescence micrographs of (a) primary hMSCs, (b) tumor MG-63, and (c) HeLa cells after 48 h of incubation with γ-Fe_2_O_3_@P(HPMA-MMAA)-Dox particles.

Different concentrations of γ-Fe_2_O_3_@PHPMA nanoparticles (0.056, 1.12, 2.78, and 5.54 μg per 200 μL of medium) were non-toxic to the cells. However, Dox-conjugated Fe_2_O_3_@P(HPMA-MMAA) nanoparticles were significantly more toxic toward human MG-63 and HeLa tumor cells compared to hMSCs. In particular, the number of dying cells under the action of these nanocomposites increased by 10–20% compared to free Dox (Figure 7). In contrast, the percentage of alive hMSCs increased by 5–25% in the presence of γ-Fe_2_O_3_@P(HPMA-MMAA)-Dox particles (Figure 8). Thus, it seems that γ-Fe_2_O_3_@PHPMA particles possess a slight cytoprotective activity towards human stem cells, but partially enhance the cytotoxic effect of Dox towards tumor cell lines, especially drug-resistant cell lines, which may be an advantage when moving to pre-clinical trials. Such an effect of the nanoparticles may be explained by their enhanced accumulation inside the cells. The particles are able to penetrate cells by the selected type of endocytosis mechanism: phagocytosis, pinocytosis, or receptor mediated endocytosis [25]. In order to check this hypothesis, cellular uptake of γ-Fe_2_O_3_@P(HPMA-MMAA)-Dox nanoparticles was analyzed by fluorescence microscopy after 48 h of incubation with primary cells (hMSCs) and human tumor cells (MG-63 and HeLa); the nanoparticles were easily engulfed and well-accumulated in the target cells, but not in the hMSC cells (Figure 9).

**Figure 10:**
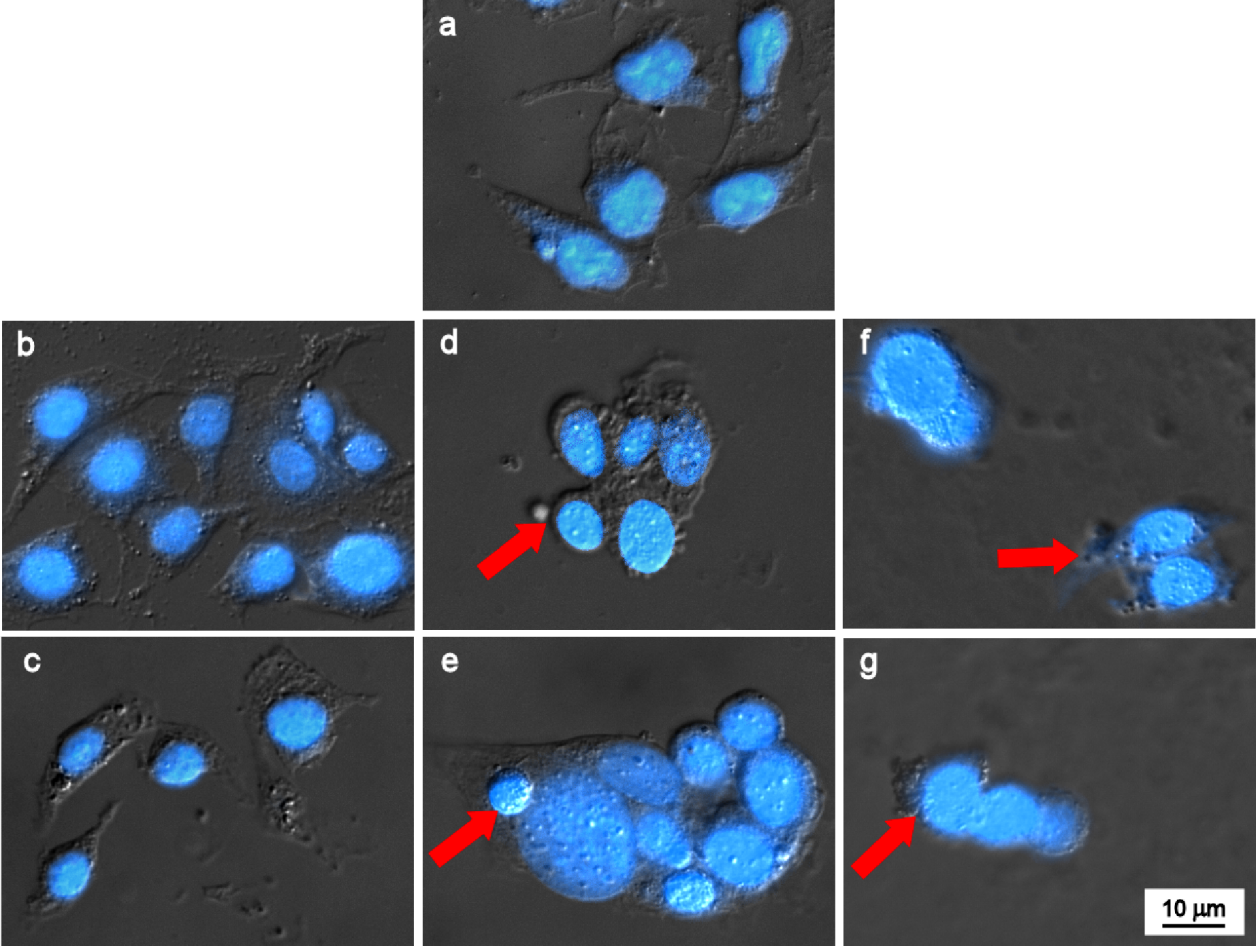
Cytomorphological determination of chromatin hypercondensation in DAPI-stained murine B16 melanoma cells incubated with (b, c) γ-Fe_2_O_3_@PHPMA (100 and 260 μg), (d, e) free Dox (5 and 13 μg), and (f, g) γ-Fe_2_O_3_@P(HPMA-MMAA)-Dox nanoparticles (100 μg/5 μg and 260 μg/13 μg, red arrows). (a) Control.

To better understand the cell-death mechanisms induced by the novel drug-delivery system, cytomorphological study of chromatin hypercondensation in DAPI-stained murine B16 melanoma cells was performed. Cells were incubated with γ-Fe_2_O_3_@PHPMA (Figure 10b,c), free Dox (Figure 10d,e), and γ-Fe_2_O_3_@P(HPMA-MMAA)-Dox nanoparticles (Figure 10f,g). Both free Dox and γ-Fe_2_O_3_@P(HPMA-MMAA)-Dox particles at two different concentrations induced apoptosis in the cells (red arrows in Figure 10d–g). It should be also noted that both γ-Fe_2_O_3_@PHPMA and P(HPMA-MMAA)-Dox nanoparticles had a tendency to adhere to the cell surface not being engulfed by them, while the cytotoxicity of Dox towards the tumor cells was preserved.

**Figure 11:**
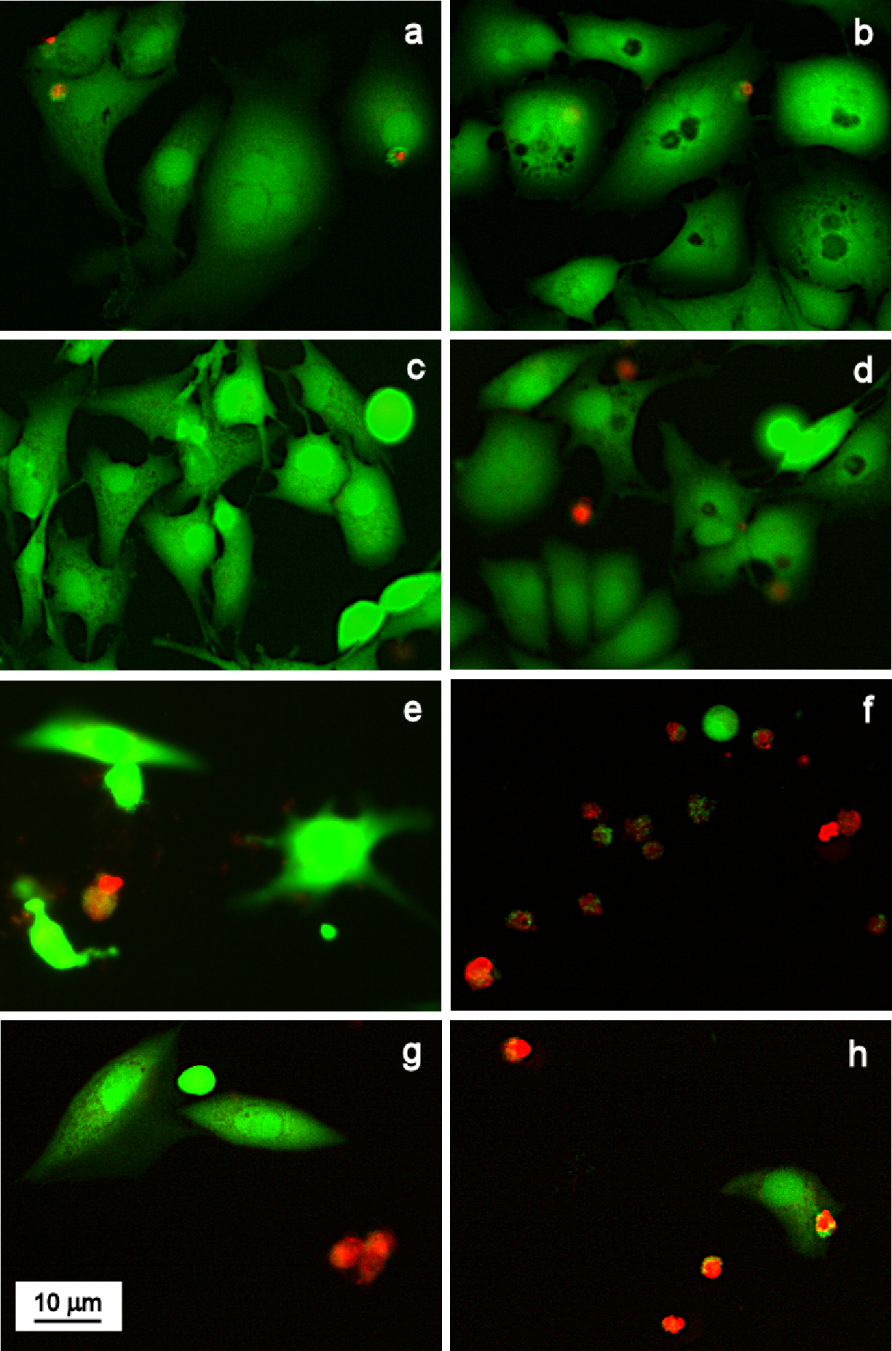
Live/dead staining of (a, c, e, g) human osteosarcoma MG-63 cells and (b, d, f, h) human cervix carcinoma HeLa cells after 48 h of incubation with (c, d) γ-Fe_2_O_3_@PHPMA (2.78 µg), (e, f) Dox (0.139 µg), and (g, h) γ-Fe_2_O_3_@P(HPMA-MMAA)-Dox particles (2.78 µg γ-Fe_2_O_3_@PHPMA + 0.139 µg P(HPMA-MMAA)-Dox). (a, b) Control.

To confirm the above results, human MG-63 and HeLa tumor cells were investigated using the live/dead assay after 48 h of incubation with γ-Fe_2_O_3_@PHPMA (2.78 µg), Dox (0.139 µg), and γ-Fe_2_O_3_@P(HPMA-MMAA)-Dox particles (2.78 µg γ-Fe_2_O_3_@PHPMA + 0.139 µg P(HPMA-MMAA)-Dox) and compared to control (Figure 11). Both free Dox and γ-Fe_2_O_3_@P(HPMA-MMAA)-Dox nanoparticles induced cell death as documented by an increased number of dead cells and decreased number of live cells (Figure 11).

## Conclusion

Superparamagnetic iron oxide nanoparticles can be manipulated using a magnetic field allowing for easy separation and/or targeted delivery in the organism [26]. In this report, citrate-treated maghemite nanoparticles and a novel PHPMA-based surface coating were used to ensure biocompatibility, minimal immunogenicity and to provide reactive functional groups for subsequent chemical conjugation with a specific drug. Dox-conjugated PHPMA was attached via hydrogen bonding between hydroxy groups on the superparamagnetic γ-Fe_2_O_3_ nanoparticle surface (citrate stabilization) and the C=O groups of the polymer. Due to the highly hydrophilic and flexible nature of PHPMA, internalization of PHPMA-modified γ-Fe_2_O_3_ particles by the cells was restricted and the particles were localized mostly on the cell surface and in the perimembranous space, where Dox retained its activity. To get comparable dose-dependent data, the amount of Dox in P(HPMA-MMAA)-Dox added to the γ-Fe_2_O_3_@PHPMA nanoparticles always corresponded to the amount of free Dox applied to the cells reaching IC_50_ level. After incubation with the cells, the Dox-free particles proved to be non-toxic. In contrast, P(HPMA-MMAA)-Dox coating on the nanoparticles enhanced the cytotoxic activity by 15–20% compared to free Dox, both for drug-sensitive and drug*-*resistant tumor cell lines, probably due to a higher affinity of the particles to the cells compared to Dox alone. This phenomenon was not observed for primary cells (hMSCs). Enhanced cytotoxicity of novel γ-Fe_2_O_3_@P(HPMA-MMAA)-Dox particles was confirmed by live/dead assay due to an increased number of dead cells and decreased number of live cells. γ-Fe_2_O_3_@P(HPMA-MMAA)-Dox nanoparticles induced apoptosis of tumor cells, as confirmed by the appearance of chromatin hypercondensation in B16 melanoma cells. The increased cytotoxicity of γ-Fe_2_O_3_@P(HPMA-MMAA)-Dox nanoparticles was due to a higher proapoptotic activity. It is supposed that the γ-Fe_2_O_3_@P(HPMA-MMAA)-Dox nanoparticles release Dox by hydrolysis of unstable hydrazone bonds in an acidic environment of the tumor cells. The mechanism of the cytotoxic effect of the γ-Fe_2_O_3_@P(HPMA-MMAA)-Dox nanoparticles is, however, still under study. It can be concluded that the newly designed γ-Fe_2_O_3_@P(HPMA-MMAA)-Dox nanoparticles are highly promising for the delivery of cancer medications into tumors, offering enhanced cell adhesion, increased apoptosis, minimal immunogenicity, lipid peroxidation, DNA damage, and reduced nonspecific toxicity. However, the obtained data indicate necessity of further in vivo studies of novel Dox-nanoparticle conjugates on experimental tumor models in mice.
